# P65 A patient-led survey into the benefits and limitations of telemedicine appointments for assessing children and young people with rheumatic conditions: Comparing European and Canadian cohorts

**DOI:** 10.1093/rap/rkac067.065

**Published:** 2022-09-28

**Authors:** Richard Beesley, Saskya Angevare, Jennifer Wilson, Wendy Costello

**Affiliations:** Juvenile Arthritis Research, Tonbridge, United Kingdom; European Network for Children with Arthritis, Geneva, Switzerland; European Network for Children with Arthritis, Geneva, Switzerland; KAISZ, Amsterdam, Netherlands; Cassie And Friends Society, Vancouver, Canada; iCAN Ireland, Bansha, Ireland; European Network for Children with Arthritis, Geneva, Switzerland

## Abstract

**Introduction/Background:**

During the COVID-19 (coronavirus) pandemic, some provision of healthcare shifted to remote, technology-assisted appointments (telemedicine). Whilst parents/carers of children and young people with rheumatic conditions have reported benefits of telemedicine, concerns remain.

This patient and parent-led project sought to understand the views of parents/carers about telemedicine, identifying the benefits and limitations of remote technology-assisted appointments, and comparing views between Canadian and European cohorts.

**Description/Method:**

An online survey was developed, translated into multiple languages and shared via social media and patient organisations, targeted at parents of children and young people with rheumatic, autoimmune and autoinflammatory conditions. Fieldwork took place in April 2021 in Europe and May 2021 in Canada. Consent was provided during enrolment.

**Discussion/Results:**

A total of 290 responses were received (133 Europe; 157 Canada; 73% female, median age 12).

Over half of respondents (53%) in Europe reported travelling over an hour to in-person appointments with their paediatric rheumatologist, compared to a significantly higher proportion of respondents in Canada (87%). Consequently, in-person appointments represent a greater time burden amongst Canadian caregivers, though both groups report appointments taking over three hours in total (51% Europe, 69% Canada).

Prior to COVID-19, most had never had a telemedicine appointment (92% Europe, 95% Canada). Since March 2020, the majority (71% Europe, 82% Canada) had at least one telemedicine appointment.

Table 1 shows the scores (1 worst, 5 best) given by parents about their telemedicine experience. Overall, most aspects scored positively (p<.05). However, parents felt telemedicine was not as good as in-person appointments.

**Key learning points/Conclusion:**

Overall respondents said they would prefer the next appointment to be in-person (82% Europe, 62% Canada, p<.05), although 31% from Canada were amenable to a combination of in-person and telemedicine-based care.

There are advantages to telemedicine, notably saving time and making appointments accessible. Families from Canada tended to view telemedicine more favourably than those from Europe, although the majority from both cohorts reported concerns about the ability to assess their child. There may be value in providing training to parents to enhance the accuracy of home-based assessments, particularly when the disease is stable. However, parents continue to report the value of in-person appointments.

**Table 1:** P65 Mean scores for a range of aspects of telemedicine (1-worst; 5-best).

	Europe	Canada	P
Easy to schedule	3.50 (3.18, 3.82) *	4.33 (4.14, 4.52) *	^†^
On time	3.22 (2.89, 3.55)	4.07 (3.85, 4.28) *	^†^
Enough time with doctor	3.51 (3.19, 3.83) *	4.24 (4.02, 4.45) *	^†^
As good as in-person visit	2.21 (1.96, 2.46) **	2.66 (2.42, 2.90) **	
Easier to see doctor	2.84 (2.55, 3.13)	3.51 (3.25, 3.77) *	^†^
Easy to sign-in	3.52 (3.22, 3.82) *	4.25 (4.06, 4.43) *	^†^
Quality of video	3.23 (2.93, 3.53)	3.87 (3.66, 4.07) *	^†^
Quality of sound	3.54 (3.26, 3.81) *	3.94 (3.75, 4.14) *	
Able to speak freely	3.61 (3.34, 3.88) *	4.05 (3.85, 4.24) *	
Able to understand doctor	3.61 (3.32, 3.90) *	4.09 (3.90, 4.28) *	
Quality of care provided	3.43 (3.12, 3.73) *	3.78 (3.56, 4.00) *	
Overall telemedicine experience	3.23 (2.91, 3.55)	3.78 (3.57, 3.99) *	^†^

* Positive score (p<.05).

** Negative score (p<.05).

† Difference between Canadian and European cohorts is statistically significant (p<.05), chi-square.

